# Clinical factors to predict flare-up in patients with inflammatory bowel disease during international air travel: A prospective study

**DOI:** 10.1371/journal.pone.0262571

**Published:** 2022-01-21

**Authors:** Jihye Park, Hyuk Yoon, Cheol Min Shin, Young Soo Park, Nayoung Kim, Dong Ho Lee

**Affiliations:** 1 Department of Internal Medicine, Institute of Gastroenterology, Yonsei University College of Medicine, Seoul, Korea; 2 Department of Internal Medicine, Seoul National University Bundang Hospital, Seongnam, Korea; 3 Department of Internal Medicine and Liver Research Institute, Seoul National University College of Medicine, Seoul, South Korea; OpenSourceResearch Organisation, DENMARK

## Abstract

**Backgrounds and aims:**

Inflammatory bowel disease (IBD) patients often experience disease flare-ups during international air travel. We aimed to identify risk factors associated with IBD flare-up during international air travel.

**Methods:**

Patients with scheduled international air travel were enrolled in the study from the Seoul National University Bundang Hospital IBD clinic. Flight information and clinical data were collected via questionnaires and personal interviews, and risk factors associated with IBD flares were determined.

**Results:**

Between May 2018 and February 2020, 94 patients were prospectively enrolled in the study (mean age, 33.0 years; males, 53.2%; mean disease duration, 56.7 months), including 56 (59.6%) with ulcerative colitis and 38 (40.4%) with Crohn’s disease. Of the 94 patients enrolled, 15 (16.0%) experienced an IBD flare-up and 79 (84.0%) remained in remission throughout travel. Logistic regression analysis revealed that high fecal calprotectin levels before travel (odds ratio [OR]: 1.001, 95% confidence interval [CI]: 1.000–1.001, *p* = 0.016), the presence of a comorbidity (OR: 6.334, 95% CI: 1.129–35.526, *p* = 0.036), and history of emergency room visit (OR: 5.283, 95% CI: 1.085–25.724, *p* = 0.039) were positively associated with disease flare-up. The previous and current use of immunomodulators and biologics, time of flight, altitude, number countries visited, travel duration, objective of visit, and previous medical consultations were not associated with disease flare-up.

**Conclusions:**

Elevated fecal calprotectin levels, history of emergency room visits, and the presence of a comorbidity predicted IBD flare-up during international air travel.

## Introduction

The incidence and prevalence of inflammatory bowel disease (IBD) has increased worldwide throughout the 21^st^ century [1]. IBD consists of chronic idiopathic refractory inflammatory disorders of the gastrointestinal tract, and includes both ulcerative colitis (UC) and Crohn’s disease (CD). UC patients present with bloody, mucoid stool, and diarrhea due to abnormalities of the mucosa of colon [2]. Abdominal pain, weight loss, and hematochezia in CD patients is typically caused by transmural inflammation of the gastrointestinal tract [3, 4]. Therapeutic targets for IBD have been developed to achieve clinical, endoscopic, and histological remission in IBD patients using aminosalicylates, steroids, immunomodulators, and various other biological agents [5, 6]. Nevertheless, IBD flare-ups may occur unexpectedly. Previous studies have identified factors capable of triggering IBD, such as nonsteroidal anti-inflammatory drugs (NSAIDs), tobacco, infections, and poor adherence to IBD treatment [7, 8].

International air travel has become popular for both business and leisure travel and has increased over time as a result of its increased degree of accessibility and the efficiency of air flight [9]. Immunocompromised patients are at greater risk of travel-related health problems due to immune system deficits [10]. IBD patients treated with immunomodulators and/or biological agents are at a high risk of acquiring infectious disease [11]. Additionally, IBD flare-ups that occur during international air travel may occur at an increased frequency as a result of diet abnormalities, the high altitude, and the limited availability of IBD medications [11]. However, comprehensive advice given prior to air travel is often overlooked in clinical practice, principally because data related to international air travel in IBD patients are scarce. Researchers previously determined that only 23% of travelers received pre-travel medical advice regarding IBD after attending an outpatient clinic [12]. In another study, 30% of travelers with IBD limited air travel and 40% of travelers reported that their choice of destination was affected because of concerns regarding access to toilets, the availability of suitable food, and the quality of medical care at the travel destination [13]. However, the study assessed the risk factors related to IBD flare-up due to air travel was scarce. Therefore, we aimed to identify risk factors associated with IBD flare-up during international air travel.

## Materials and methods

### Patients

Initially, we prospectively enrolled IBD patients for whom international air travel was scheduled and clinical remission was maintained from an IBD outpatient clinic at Seoul National University Bundang Hospital (Seongnam, Republic of Korea). Additionally, patients who reported that they had travelled internationally at the first outpatient visit that followed the trip were also included in the study. Clinical remission was defined as a partial Mayo score ≤ 1 in UC patients and a CD activity index (CDAI) score < 150. Patients were excluded from the study if their symptoms were worsening or if laboratory data were incomplete. For this study, we conducted a questionnaire-based survey of IBD patients who participated in international air travel. We used IBD cohort data that included extensive medical records from each clinic visit.

### Data collection and definitions

Questionnaires and personal interviews used to provide information regarding international air travel were obtained from all patients at the time of study enrollment. Information gathered included the flight time, number of countries visited, number of cities visited, travel duration, objective of the visit (leisure or business), presence of a pre-travel medical consultation, and previously prescribed medication. The consolidated criteria for reporting qualitative studies (COREQ) checklist was used to guide the report of the methods section. We investigated baseline clinical characteristics, including age, sex, a history of smoking, extraintestinal manifestation, comorbidity, IBD subtype, disease extent and activity, previous and current medications, a history of hospitalization, a history of an emergency room visit, prior abdominal surgery, and prior anal surgery. Comorbidities were defined as conditions with a Charlson comorbidity index ≥ 1. The partial Mayo score for UC and the CDAI for CD were investigated within a 3-month period that preceded air travel. Pre-travel laboratory data, including C-reactive protein and fecal calprotectin levels, were also obtained within the 3-month period that preceded air travel. We defined the countries which has a high risk of experiencing traveler’s diarrhea in accordance with a previous report [14].

The primary outcome of this study was the IBD flare-up during international air travel. We compared the group of patients that experienced an IBD flare-up with the group that remained in remission during international air travel. Patients that experienced an IBD flare-up experienced the worsening of symptoms (increased bowel frequency with rectal bleeding in UC patients and increased bowel frequency with abdominal pain in CD patients) and/or were prescribed medication during air travel. An assessment of IBD was performed during the first outpatient visit that followed air travel [15].

### Statistical analysis

Medians and quantiles were calculated for all continuous variables, and proportions (%) were calculated for all categorical variables, as appropriate. Because data were not normally distributed, the Mann-Whitney U test was used to assess continuous variables. The Fisher’s exact test was used to assess categorical variables. Multivariate logistic regression analyses were carried out to identify independent risk factors of IBD flare-up that were associated with international air travel in IBD patients, with adjustments performed for various confounders. Covariates with a *p* value < 0.1 in the univariate analysis were subjected to multivariable Cox proportional hazards analysis. All statistical analyses were performed using the Statistical Package for Social Sciences (SPSS version 25.0; SPSS Inc., Armonk, NY, USA). Values of *p* < 0.05 were considered statistically significant.

### Ethical considerations

This study was approved by the Institutional Review Board of Seoul National University Bundang Hospital (IRB No: B-1701-380-303). Written informed consent for the use of medical records was provided by all participants.

## Results

### Patient characteristics

From May 2018 to February 2020, a total of 94 patients who visited the IBD clinic of Seoul National University Bundang Hospital were enrolled in the study. Baseline patient characteristics are summarized in **Table 1**. The median age of patients was 33.0 years, and 50 patients (53.2%) were male. The median disease duration was 55.0 months. In this study, 59.6% of the patients enrolled were UC patients and 40.4% were CD patients. Personal interviews revealed that 33 patients (35.1%) were currently using mono or combined biological therapy and 24 patients (25.5%) reported the use of immunomodulators as monotherapy. Eleven patients (11.7%) had a history of abdominal surgery due to IBD.

**Table 1:** Patient characteristics (N = 94).

Variables	Total (N = 94)	Remission (N = 79)	Flare-up (N = 15)	*p* value
Age	33.0 (26.0–48.0)	33.0 (26.0–48.0)	40.0 (26.0–48.0)	0.691
Sex, Male	50 (53.2%)	41 (51.9%)	9 (60.0%)	0.564
Current smoking (vs. past or never smoking)	9 (9.6%)	7 (8.9%)	2 (13.3%)	0.632
Extraintestinal manifestation	12 (12.8%)	12 (15.2%)	0 (0.0%)	0.203
Presence of comorbidity	7 (7.4%)	4 (5.1%)	3 (20.0%)	0.078
Disease duration (months)	55.0 (16.0–76.0)	53.1 (16.7–72.4)	65.1 (15.0–119.5)	0.403
UC (vs. CD)	56 (59.6%)	46 (58.2%)	10 (66.7%)	0.541
UC, Extent (N = 56)				0.212
UC proctitis	18 (32.1%)	17 (37.0%)	1 (10.0%)	
Left-sided colitis	10 (17.9%)	7 (15.2%)	2 (30.0%)	
Pancolitis	28 (50.0%)	22 (47.8%)	6 (60.0%)	
CD, Age (N = 38)				0.642
A1 (< 17)	6 (15.8%)	5 (15.2%)	1 (20.0%)	
A2 (17~40)	27 (71.1%)	23 (69.7%)	4 (80.0%)	
A3 (> 40)	5 (13.2%)	5 (15.2%)	0 (0.0%)	
CD, Location (N = 38)				0.522
L1 (Ileum)	6 (15.8%)	6 (18.2%)	0 (0.0%)	
L2 (Colon)	1 (2.6%)	1 (3.0%)	0 (0.0%)	
L3 (Ileuocolonic)	31 (81.6%)	28 (78.8%)	5 (100.0%)	
CD, Behavior (N = 38)				0.527
B1 (Inflammatory)	22 (57.9%)	18 (54.5%)	4 (80.0%)	
B2 (Stricturing)	3 (7.9%)	3 (9.1%)	0 (0.0%)	
B3 (Penetrating)	13 (34.2%)	12 (36.4%)	1 (20.0%)	
Previous medical history				
Corticosteroid or immunomodulator	69 (73.4%)	58 (73.4%)	11 (73.3%)	1.000
Biologics	17 (18.1%)	13 (16.5%)	4 (26.7%)	0.462
Current medical history				0.845
5-ASA	37 (39.4%)	31 (39.2%)	6 (40.0%)	
Immunomodulator	24 (25.5%)	21 (26.6%)	3 (20.0%)	
Biologics	33 (35.1%)	27 (34.2%)	6 (40.0%)	
Previous hospitalization	51 (54.3%)	42 (53.2%)	9 (60.0%)	0.626
Previous emergency room visit	56 (59.6%)	44 (55.7%)	12 (80.0%)	0.093
Previous abdominal surgery	11 (11.7%)	10 (12.7%)	1 (6.7%)	1.000
Partial Mayo score (N = 56)	2.0 (0.0–2.0)	1.0 (0.0–2.0)	2.0 (0.8–3.0)	0.254
Crohn’s disease activity index (N = 38)	43.0 (5.4–62.8)	39.0 (4.0–61.4)	48.0 (46.0–73.4)	0.134
Laboratory findings				
C-reactive protein (mg/L)	0.10 (0.03–0.34)	0.08 (0.03–0.33)	0.12 (0.04–0.36)	0.652
Fecal calprotectin (ug/mg)	288.0 (75.2–727.9)	254.5 (70.0–574.0)	762.0 (238.5–1898.1)	0.028

Variables are expressed as median (IQR) or n (%).

*p value for comparing remission and flare-up groups.

UC, ulcerative colitis; CD, Crohn’s disease; TNF, tumor necrosis factor.

A total of 15 patients (16.0%) experienced an IBD flare-up during international air travel. There were no significant differences between patients who did and did not experience a flare-up regarding smoking history, the Charlson comorbidity index, disease extent and severity, the previous and current medical history, previous hospitalization, emergency room visit, abdominal and anal surgery history (**Table 1**). Pre-travel C-reactive protein levels of both groups were similar. Pre-travel fecal calprotectin levels were elevated in patients of the IBD flare-up group compared to those of the remission group (762.0 ug/mg vs. 254.5 ug/mg, respectively, *p* = 0.028; **Table 1**).

### International air travel information

The median flight duration was 12.0 hours (interquartile range [IQR], 5.5–24.0 hours) and the altitude of the city to which patients traveled was not high (median, 19.0 m; IQR, 4.4–24.0 m; **Table 2**). The median number of countries and cities visited was 1.0 (IQR, 1.0–1.0) and the median duration of travel was 8.0 days (IQR, 4.0–13.3 days). No differences in international air travel information were recorded from patients in the IBD flare-up group and the remission group. The rate of visits to areas associated with traveler’s diarrhea was high (47.9%), but no differences between the two groups were observed. The percentage of patients who traveled for leisure purposes was 76.6% and that for patients who traveled for business purposes was 23.4%, and no difference between the two groups were observed. Pre-travel medical consulting was provided to 66 patients (70.2%), and 26 patients (27.7%) received an additional prescription in preparation for travel. No differences between the assessed groups were observed regarding the receipt of additional prescriptions.

**Table 2:** Fight information in patients with inflammatory bowel disease (N = 94).

Variables	Total (N = 94)	Remission (N = 79)	Flare-up (N = 15)	*p* value
Flight time (hours)	12.0 (5.5–24.0)	12.0 (8.0–24.0)	16.0 (4.0–28.0)	0.799
Altitude above sea level (m)	19.0 (6.0–66.3)	19.0 (6.0–70.0)	23.0 (6.0–65.0)	0.885
Number of country	1.0 (1.0–1.0)	1.0 (1.0–1.0)	1.0 (1.0–1.0)	0.093
Number of city	1.0 (1.0–1.0)	1.0 (1.0–2.0)	1.0 (1.0–1.0)	0.083
Travel duration (days)	8.0 (4.0–13.3)	8.0 (4.0–14.0)	9.0 (4.0–10.0)	0.864
Visit of country with high risk traveler’s diarrhea	45 (47.9%)	38 (48.1%)	7 (46.7%)	1.000
Objective for visit				0.109
Leisure	72 (76.6%)	63 (79.7%)	9 (60.0%)	
Bussiness	22 (23.4%)	16 (20.3%)	6 (40.0%)	
Pre-travel medical consulting	66 (70.2%)	57 (72.2%)	9 (60.0%)	0.367
Previous additional prescription	26 (27.7%)	19 (24.1%)	7 (46.7%)	0.073

Variables are expressed as median (IQR) or n (%).

*p value for comparing remission and flare-up groups.

### Risk factors related to IBD flare-up during international air travel

Multivariate logistic regression analyses were carried out to determine risk factors associated with international air travel. The presence of comorbidity (odds ratio [OR]: 6.334, 95% confidence interval [CI]: 1.129–35.526, *p* = 0.036), a history of emergency room visit (OR: 5.283, 95% CI: 1.085–25.724, *p* = 0.039), and fecal calprotectin levels (OR: 1.001, 95% CI: 1.000–1.001, *p* = 0.016) were positively associated with the occurrence of IBD flare-up during international air travel (**Table 3**).

**Table 3:** Logistic regression analysis for IBD flare-up related to international air travel in inflammatory bowel disease patients.

Variables	Univariate analysis			Multivariate analysis		
	OR	95% CI	*p value	OR	95% CI	*p value
Age	1.001	0.963–1.040	0.968			
Males	0.719	0.234–2.212	0.565			
Smoking	0.632	0.118–3.387	0.592			
Presence of comorbidity	4.687	0.931–23.597	0.061	**6.334**	**1.129–35.526**	**0.036**
Disease duration	1.003	0.995–1.011	0.486			
CD versus UC	0.697	0.218–2.230	0.543			
Previous medical history						
Corticosteroid or immunomodulator	0.996	0.286–3.470	0.995			
Biologics	1.846	0.508–6.705	0.541			
Current medical history						
5-ASA	1.000	(reference)				
Immunomodulator	0.738	0.166–3.283	0.690			
Biologics	1.148	0.331–3.982	0.828			
Previous hospitalization	1.486	0.496–4.446	0.479			
Previous emergency room visit	3.182	0.833–12.161	0.091	**5.283**	**1.085–25.724**	**0.039**
Previous abdominal surgery	1.948	0.232–16.372	0.539			
Laboratory findings						
C-reactive protein (mg/L)	1.017	0.737–1.404	0.918			
Fecal calprotectin (ug/mg)	1.001	1.000–1.002	0.058	**1.001**	**1.000–1.001**	**0.016**
Flight time (hours)	1.011	0.956–1.070	0.704			
Altitude above sea level (m)	0.998	0.993–1.004	0.514			
Number of country	0.000	0.000–0.000	0.997			
Number of city	0.319	0.061–1.674	0.177			
Travel duration (days)	1.000	0.964–1.038	0.991			
Visit of country with high risk traveller’s diarrhea	0.944	0.312–2.854	0.919			
Objects for visit (business vs leisure)	2.625	0.815–8.455	0.106			
Pre-travel medical consulting	0.579	0.184–1.818	0.349	** **	** **	** **

*p value for comparing patients with remission and patients with relapse after travel

OR, odds ratio; CI, confidence interval; CD, Crohn’s disease; UC, ulcerative colitis.

## Discussion

IBD flare-ups during international air travel were experienced in 15 of the 95 total patients considered in our study (16.0%), which is a flare rate similar to that which was reported previously (15.1%) [16]. This is the first study to demonstrate that fecal calprotectin levels are associated with IBD flare-ups. An elevation in fecal calprotectin levels by 100 ug/g increased IBD flare risk 10%. Patients with high fecal calprotectin levels before air travel had an elevated risk of IBD flare-up during travel, despite their clinical remission status. The recently developed IBD treatment paradigm involves treatment to control intestinal inflammation using a targeted strategy [17, 18]. The main principle of the paradigm involves the regular assessment and monitoring of disease activity using various objective tests. Findings reported here have the potential to facilitate the implementation of this concept. Our study demonstrated that throughout remission, monitoring fecal calprotectin levels may predict the occurrence of an IBD flare-up during international air travel. The use of this data would allow us to advise IBD patients in clinical remission in order to reduce the occurrence of disease flare-ups during air travel.

A total of 57 patients (60.6%) were treated with immunomodulators and/or biological agents in present study. Of the 33 patients who maintained clinical remission with biological agents assessed here, 23 were provided subcutaneous injections during international air travel. Here, the patient’s current medical history did not affect the occurrence of disease flare-ups during international air travel. *Ben-Horin* et al. reported that IBD patients treated with immunomodulators had a similar risk of illness to immunosuppressed patients, which is a finding that was similar to observations reported here [16]. Theoretically, the use of immunomodulators or biological agents should diminish the immune status of patients and enhance the occurrence of opportunistic infection. The vaccination and pre-travel personal hygiene counseling of patients prior to international air travel is very important [19]. Nevertheless, in reality, the maintenance of immunomodulators or biological agents did not enhance the occurrence of disease flare-up. Therefore, it might be more desirable to maintain remission status with these drugs during air travel. At least 4 out of 17 patients likely injected adalimumab while they were out of the country, because they traveled for more than two weeks.

A history of emergency room (ER) visit was associated with IBD flare-up during international air travel. *Ben-Horin* et al. reported that prior hospitalizations and the number of flares were positively associated with travel-related illness in IBD patients [16]. In other words, in patients who maintained remission status at an outpatient visit without ER visit, the risk of IBD flare-up was low during international air travel. Therefore, we could provide pre-travel counseling and additional prescriptions to IBD patients with a history that included an ER visit.

In the present study, the occurrence of an IBD flare-up during international air travel was 7.2 times greater in patients with comorbid diseases than those without comorbidities. *Roman e*t al. reported that, in IBD patients, a comorbidity, especially cardiovascular morbidity, was associated with increased overall morbidity [20]. Recently, the knowledge of the presence of a comorbid condition has been reported to be crucial for management of IBD patients because comorbidities can alter disease activity, disease course, drug choice, and QOL [21]. In accordance with our results, *Hochberg* et al. described high-risk international travelers as immunocompromised travelers, those with medical comorbidities, and pregnant women [22].

A previous study performed in Switzerland demonstrated that high-altitude journeys increase the occurrence of IBD flare-ups [23]. High-altitude journeys were defined as stays at altitudes more than 2,000 m above sea level. Among destinations studied, the Swiss Alps was the most frequently visited travel destination. Hypoxia could aggravate IBD pathogenesis by activating hypoxia-inducible factors, translocating nuclear factor-kB and toll-like receptor, inducing the hyperactivation of effector immune cells, and promoting intestinal inflammation [24]. The median altitude above sea level of destinations assessed here was lower than that of the previous study, and no difference in median altitude level was observed between the remission and IBD flare-up group. Furthermore, the flight time, number of countries visited, number of cities visited, and travel duration were not associated with IBD flare-up. Therefore, international air travel itself was not determined to be associated with IBD flare-ups.

European Crohn’s and Colitis Organization developed an IBD passport, which is a website that was created to provide evidence-based travel advice and education for individual IBD patients [25, 26]. Vaccination guidelines suggest practical strategies that can be used by IBD patients to promote health while traveling [27, 28]. However, pre-travel medical consulting and vaccination rates remain low in the clinical setting [12, 27]. To improve QOL of IBD patients throughout international air travel, the disease status of patients should be evaluated using fecal calprotectin levels, and their previous ER visit history and the occurrence of comorbidities should be reviewed.

This study has several limitations. First, the number of patients enrolled the study was relatively small. Therefore, a prospective study with larger sample size will be needed to confirm our results after the Coronavirus disease era. Second, the study was mainly conducted using a prospective design that included patients who were enrolled before traveling internationally, but some patients enrolled after travel were also included. However, pre-travel medical consulting was not a predictive factor for IBD flare-up during air travel.

In conclusion, fecal calprotectin levels, the occurrence of a previous ER visit, and the presence of comorbidities were determined to predict IBD flare-ups during international air travel.

## Supporting information

S1 Appendix IBD air travel questionnaires. (DOCX)

S2 Appendix Consolidated criteria for reporting qualitative studies (COREQ): 32-item checklist. (DOCX)

## Abbreviations

IBD: Inflammatory bowel disease
UC: Ulcerative colitis
CD: Crohn’s disease
NSAIDs: nonsteroidal anti-inflammatory drugs
IQR: interquartile range
OR: odds ratio
CI: confidence interval
QOL: quality of life
ER: emergency room
