# Supplementary material for: Clinical factors to predict flare-up in patients with inflammatory bowel disease during international air travel: A prospective study
Source: PLoS One. 2022 Jan 21;17(1):e0262571. doi: 10.1371/journal.pone.0262571 (PMC8782361; doi:10.1371/journal.pone.0262571)
Supplement: S1 Appendix — (DOCX) [file pone.0262571.s001.docx]

**IBD air travel Questionnaires**

**Personal details**

| **Item** |  |
| --- | --- |
| Name |  |
| Sex |  |
| Date of birth |  |

**Air travel description**

| **Item** |  |
| --- | --- |
| Date of departure |  |
| Date of return |  |
| List of countries to visit |  |
| List of cities to visit |  |
| Flight time (one-way) |  |
| What was the purpose of your traveling? | 1) Leisure  2) Business |

**Pre-travel and post-travel questionnaire**

| Have you taken a pre-travel medical consultation? |  |
| --- | --- |
| Do you have previously prescribed medication for air travel? |  |
| Have you ever experienced flare-ups of your disease during your travel? |  |
| Write downany further information that might be relevant. |  |
